# Pharmacokinetics and Metabolism of Acetyl Triethyl Citrate, a Water-Soluble Plasticizer for Pharmaceutical Polymers in Rats

**DOI:** 10.3390/pharmaceutics11040162

**Published:** 2019-04-03

**Authors:** Hyeon Kim, Young Seok Ji, Shaheed Ur Rehman, Min Sun Choi, Myung Chan Gye, Hye Hyun Yoo

**Affiliations:** 1 Institute of Pharmaceutical Science and Technology and College of Pharmacy, Hanyang University, Ansan, Gyeonggi-do 15588, Korea; kimhyeon2000@nate.com (H.K.); wldudtjr23@hanyang.ac.kr (Y.S.J.); chm2456@hanyang.ac.kr (M.S.C.); 2 Department of Pharmacy, COMSATS, Institute of Information Technology, Lahore, Punjab 54000, Pakistan; dr.shaheedmarwat@yahoo.com; 3 Department of Life Science, Institute of Natural Sciences, Hanyang Uuiversity, Seoul 04763, Korea

**Keywords:** acetyl triethyl citrate, plasticizer, pharmaceutical polymers, pharmacokinetics, metabolism

## Abstract

Acetyl triethyl citrate (ATEC) is a water-soluble plasticizer used in pharmaceutical plasticized polymers. In this study, the pharmacokinetics and metabolism of ATEC were investigated using liquid chromatography–tandem mass spectrometry (LC–MS/MS) in rats. Plasma protein precipitation with methanol was used for sample preparation. For chromatographic separation, a C18 column was used. The mobile phases consisted of 0.1% formic acid and 90% acetonitrile, and gradient elution was used. The following precursor-product ion pairs were selected for reaction monitoring analysis: 319.1 *m*/*z* → 157 m/z for ATEC and 361.2 *m*/*z* → 185.1 m/z for tributyl citrate (internal standard) in positive ion mode. The LC–MS/MS method was fully validated and successfully applied to a pharmacokinetic study of ATEC in rats. The pharmacokinetic study showed that the volume of distribution and mean residence time of ATEC were higher after oral administration than after intravenous administration, pointing to extensive first-pass metabolism and distribution in tissue. In addition, the plasma concentration profile of the postulated metabolites of ATEC was investigated in plasma, urine, and feces. The resulting data indicated that ATEC was extensively metabolized and excreted mainly as metabolites rather than as the parent form. The developed analytical method and the data on the pharmacokinetics and metabolism of ATEC may be useful for understanding the safety and toxicity of ATEC.

## 1. Introduction

Plasticized polymers are substantial in pharmaceutical technology. For example, they can be used as coating materials of dosage forms, free membranes of pharmaceutical films, polymeric membranes of transdermal system, matrix systems for extended release formulations, or microparticles [1]. Plasticizers play important roles in diverse pharmaceutical plasticized polymers by improving their flexibility and processability [1].

Acetyl triethyl citrate (ATEC) is one of the plasticizers used in pharmaceutical plasticized polymers [1]. It is an aliphatic ester of citric acid which is a clear oily liquid with essentially no odor. In pharmaceutical polymers, ATEC is used as a hydrophilic plasticizer in the coating of press-coated tablets that are composed of hydroxypropyl methylcellulose acetate succinate or enteric polymer films consisting of polymethacrylic acid methylmethacrylate [1,2,3,4,5]. ATEC is also used in protein films composed of whey protein or sunflower protein for buccal and sublingual films or particulate systems [1]. Given the uses of ATEC in various pharmaceutical formulations, the safety and toxicity of ATEC in the body should be thoroughly evaluated. 

There have been some toxicology reports on ATEC. In terms of acute oral toxicity, the LD50 for ATEC was reported to be approximately 7 mL/kg in rats and cats [6]. ATEC doses of 6–12 mL/kg caused progressive lowering of the blood pressure [7]. In a study of short-term oral toxicity, feeding rats a diet containing 1–4 g/kg of ATEC for 6 weeks did not affect growth or induce toxicity [6]. The same study reported no adverse effects of ATEC on blood cells [6]. In the developmental toxicity test with *Xenopus laevis* embryos, the 96 h EC50 for malformation of ATEC was 413.8 mg/L, and the 96 h lowest observed effective concentration for malformation of embryos was 363.5 mg/L [8]. These results indicate that the teratogenic potential of ATEC would be negligible, and its developmental toxicity may also be quite low. In Hershberger assays, ATEC did not show any significant androgenic or antiandrogenic activities [9]. Another study also indicated that ATEC has no effects on estrogen or antiestrogen activity or steroidogenesis. These reports suggest that ATEC may not have endocrine-disrupting activities [10]. Therefore, ATEC is considered relatively safe on the basis of the toxicological data published thus far.

However, concerns have been raised about potential blood pressure lowering effects of ATEC [7]. In addition, acetyl tributyl citrate (ATBC), which is structurally similar to ATEC, was shown to induce intestinal cytochrome P450 3A4 and affect female reproduction at a low dose [11]. Therefore, the safety of ATEC should be further investigated, and human exposure to ATEC needs to be monitored. However, the information on the pharmacokinetics of ATEC is still limited.

Analytical methods for ATEC include the use of infrared spectroscopy, nuclear magnetic resonance spectroscopy [12], and mass spectroscopy [13]. Thin-layer chromatography [14], gas chromatography [15,16], and high-performance liquid chromatography [17] have also been used for ATEC analysis. However, no studies have reported the use of liquid chromatography–tandem mass spectrometry (LC–MS/MS) for ATEC analysis. Furthermore, at present, there are no analytical methods for the determination of ATEC in biological samples.

In this study, we developed a bioanalytical method for the determination of ATEC in rat plasma using LC–MS/MS. On the basis of the developed method, we investigated the pharmacokinetic properties of ATEC in rats.

## 2. Materials and Methods 

### 2.1. Chemicals and Materials

Acetyl triethyl citrate (ATEC, >98%), tributyl citrate (TBC, internal standard), and phenylmethylsulphonyl fluoride (PMSF) were purchased from Sigma-Aldrich (St. louis, MO, USA). HPLC-grade methanol (MeOH) was purchased from J.T. Baker (Phillipsburg, NJ, USA). Water was prepared using a Milli-Q purification system (Millipore, Bedford, MA, USA). Blank plasma was collected in a heparin tube from Male Sprague-Dawley rats. All other chemicals and solvents were of analytical grade.

### 2.2. Preparation of Calibration and Quality Control (QC) Standards

A stock solution of ATEC was prepared to yield a concentration of 5 mg/mL in 100% acetonitrile (ACN). The solution was stored at −20 °C and brought back to normal room temperature before use. Working standard solutions were prepared by diluting the ATEC stock solution with 100% of ACN to final concentrations ranging from 0.1 µg/mL to 20 µg/mL. A stock solution (5 mg/mL) of internal standard (IS) TBC was prepared in 100% ACN and further diluted to a concentration of 100 ng/mL in the ACN. Blank rat plasma (90 µL) was treated with 2.5 μL of 1 M PMSF, and the working standard solutions (10 µL) were added to yield calibration standards of 10, 50, 100, 200, 500, 1000, and 2000 ng/mL. QC samples were prepared in the same way as the calibration standards, having final concentrations of 10, 30, 300, and 1600 ng/mL. The QC samples and calibration standards were kept frozen (−20 °C) until use. For protein precipitation, three volumes of the IS solution were added to calibration standards and QC samples, vortexed for 30 secs, and then centrifuged for 5 min at 10,000 × *g* and 4 °C. The supernatant was taken for LC–MS/MS analysis.

### 2.3. Validation of the Analytical Method 

To determine the specificity of the assay, chromatograms of blank and standard-spiked plasma samples were compared and evaluated using six different batches of blank plasma. Calibration curves were constructed by plotting the ratios of the peak area from the analyte to that from the IS versus the analyte concentrations. The calibration curve was calculated by linear least-squares regression. The lower limit of quantification (LLOQ) was defined as the lowest concentration with a relative standard deviation (RSD) <20% and with accuracy of 80–120%. The precision and accuracy of the method were evaluated by repeated analyses of QC samples (*n* = 5 for intra-day and *n* = 3 for inter-day) at concentrations of 10, 30, 300, and 1600 ng/mL. Matrix effects were evaluated by comparing the peak area of the post-spiked extracted sample with that acquired using a neat solution, and the extraction recovery was measured by comparing the peak area of the pre-spiked extracted sample with that of the post-spiked extracted sample. To determine process efficiency, the peak area in the neat solution was compared with that in the pre-spiked extracted sample. The spiked extracted samples were prepared using pooled blank plasma from six different batches. Bench-top, freeze-and-thaw, long-term, and postpreparative stability studies were also performed to evaluate the stability of ATEC. The methods used in the validation procedures were based on the “Guidance for Industry: Bioanalytical Method Validation” [18]. 

### 2.4. Pharmacokinetics Study in Rats

Male Sprague-Dawley rats aged 8 weeks (weight, approximately 250–290 g) were purchased from Orient Bio (Seongnam, Korea) and housed in a temperature- (23 ± 3 °C) and moisture-controlled (55 ± 15% relative humidity) room under a 12 h light–dark cycle, with access to food and water ad libitum. The rats fasted for 12 h before treatment and had free access to water. The rats were anesthetized with Zoletil 50 (Virbac, Carros, France) and Rompun^®^ (Bayer, Leverkusen, Germany), and a polyethylene tube was surgically implanted into the carotid artery one day before the pharmacokinetic study. The cannulae were fixed to the head and neck, and the rats were allowed to move freely during the experiment. Dosing solutions were prepared in 30% poly-ethylene glycol (PEG) at a concentration of 10 mg/mL for intravenous (i.v.) administration and 500 mg/mL for oral (p.o.) administration. The rats were administered ATEC intravenously (10 mg/kg) and orally (500 mg/kg). Blood samples (200 μL) were collected from the carotid artery in tubes containing 1 μL of heparin (5000 IU) and 5 μL of an esterase inhibitor, PMSF (1M). The collection time points were 1 min, 2 min, 5 min, 10 min, 15 min, 30 min, 1 h, 2 h, 4 h, 6 h, 10 h, and 24 h after the i.v. injection and 5 min, 15 min, 30 m, 45 min, 1 h, 2 h, 4 h, 6 h, 8 h, 10 h, 24 h, and 36 h after p.o. administration. The collected blood samples were centrifuged at 10,000× *g* and 4 °C for 5 min, and the supernatant plasma was collected. 

To determine the metabolic profile of ATEC, the rats were placed in metabolic cages for 5 days after oral dosing (500 mg/kg). Urine and feces were collected 10 h, 1 day, 2 day, 3 day, 4 day, and 4.3 day after oral administration. The collected samples were kept frozen at −20 °C until use. All animal procedures were approved by the Institutional Animal Care and Use Committee of Hanyang University (2016-0235A).

### 2.5. Sample Preparation

The plasma and urine (50 μL) samples were placed in separate 1.5 mL tubes and treated with 150 μL IS (100 ng/mL of TBC in MeOH) for protein precipitation. The feces were homogenized with three volumes of ACN and centrifuged at 2000× *g* for 5 min. The supernatant (100 μL) was transferred to a tube, and 100 μL of IS was added. The tubes were vortex-mixed and centrifuged at 10,000× *g* and 4 °C for 5 min. The supernatant was placed in a HPLC vial for LC–MS/MS or liquid chromatography–quadrupole time-of-flight–mass spectrometry (LC–QTOF–MS) analysis.

### 2.6. Pharmacokinetics Analysis 

The pharmacokinetic parameters of the rat plasma samples were determined using the Phoenix WinNonlin Enterprise Program, v5.3 (Pharsight Inc., St. Louis, MO, USA) using a noncompartmental statistical model. The maximum plasma concentration (*C*_max_) and the time to reach the *C*_max_ were obtained directly from the experimental data. The elimination half-life (*t*_1/2_) was calculated as 0.693/λz, where λz is the elimination rate constant calculated from the terminal linear portion of the logged plasma concentration–time curve. To determine absolute bioavailability, the plasma drug concentration versus time plot for the drug dose after both i.v. and p.o. administrations was calculated from the experimented AUC values. The initial concentration in the i.v. administration, distribution volume based on the terminal phase (Vz), and total body clearance were obtained from the experimental data, processed by WinNonlin (ver. 5.3) software. 

### 2.7. Metabolic Stability Assay 

ATEC was incubated with pH 7.4 phosphate buffered saline at 37 °C for 0, 5, 15, 30, 60, and 120 min; with rat plasma at 37 °C for 0, 5, 15, 30, and 60 min; and with rat liver microsomes at 37 °C for 0, 1, 5, 15, 30, and 60 min. The detailed procedures have been described elsewhere [19,20]. After incubation, the sample was prepared as described in Section 2.5. 

### 2.8. LC–MS/MS

**Figure 1:**
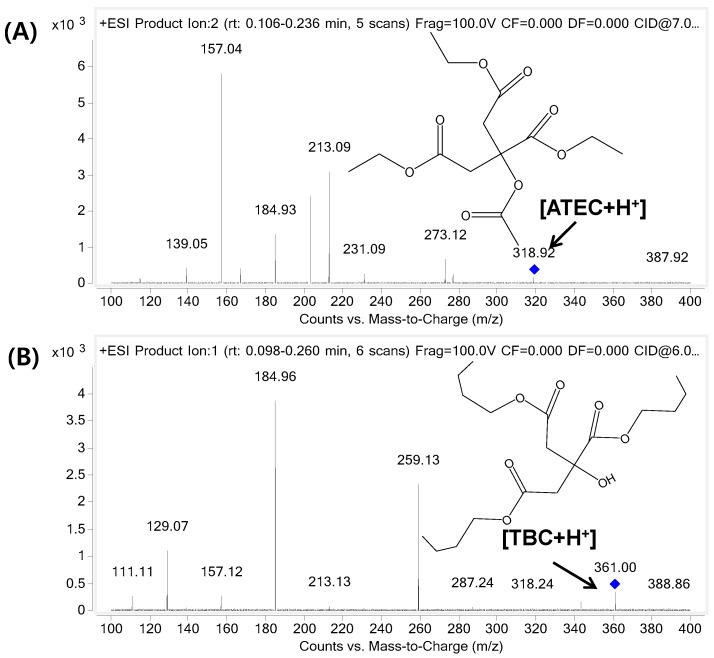
Product ion spectrum of (**A**) Acetyl triethyl citrate (ATEC) and (**B**) tributyl citrate internal standard (TBC IS).

An Agilent 1260 infinity HPLC system coupled with a 6460-triple quadrupole mass spectrometer (Agilent Technologies, Santa Clara, CA, USA) and an electrospray ionization source was used for quantitative analysis of the analytes. For chromatographic separation, a Phenomenex Kinetex C18 (2.1 mm × 50 mm, 2.1 μm) column with a thermostatically controlled column temperature at 30 °C was used. The mobile phases consisted of 0.1% formic acid in DW (solvent A) and 0.1% formic acid in 90% ACN (solvent B), with a gradient elution at a flow rate of 0.3 mL/min. The gradient elution was initiated with 10% of mobile phase B, increased to 100% of B for 0.6 min, and held for 2 min, followed by re-equilibration to the initial condition for 4 min. The total run time was 6 min, and the injection volume was 5 µL. For selected reaction monitoring analyses, the following precursor-product ion pairs were used: 319.1 *m*/*z* → 157 *m*/*z* for ATEC and 361.2 *m*/*z* → 185.1 m/z for the IS in the positive ion mode. The fragmentor voltages were 70 V and 104 V, and the collision energies were 10 V and 12 V for ATEC and IS, respectively. The product ion spectra of ATEC and TBC and their chemical structures are presented in Figure 1.

### 2.9. LC–QTOF MS 

For metabolite profiling, the samples were analyzed by an LC–QTOF–MS instrument consisting of an Agilent 1260 HPLC system and an Agilent6530 Accurate-Mass Q-TOF system (Agilent Technologies, Santa Clara, CA, USA). A Waters Xterra C18 column (2.1 mm × 150 mm, 3.5 μm) was used, and the mobile phase consisted of 0.1% formic acid (solvent A) and 0.1% formic acid in 90% ACN (solvent B). The flow rate was 0.3 mL/min. Gradient elution was performed as follows: initially, 10% B; 0–15 min, 10–50% B; 15–18 min, 50–70% B; 18–20 min, 70–90% B; 20–21 min, 90% B; 21–21.1, 90–10% B; 21.1–27, 10% B. An electrospray ionization source was used, and mass detection was performed in the positive ion mode. The capillary voltage was 4.0 kV, and the fragment energy was 150 V.

## 3. Results and Discussion

### 3.1. Analytical Method Validation 

#### 3.1.1. Selectivity

**Figure 2:**
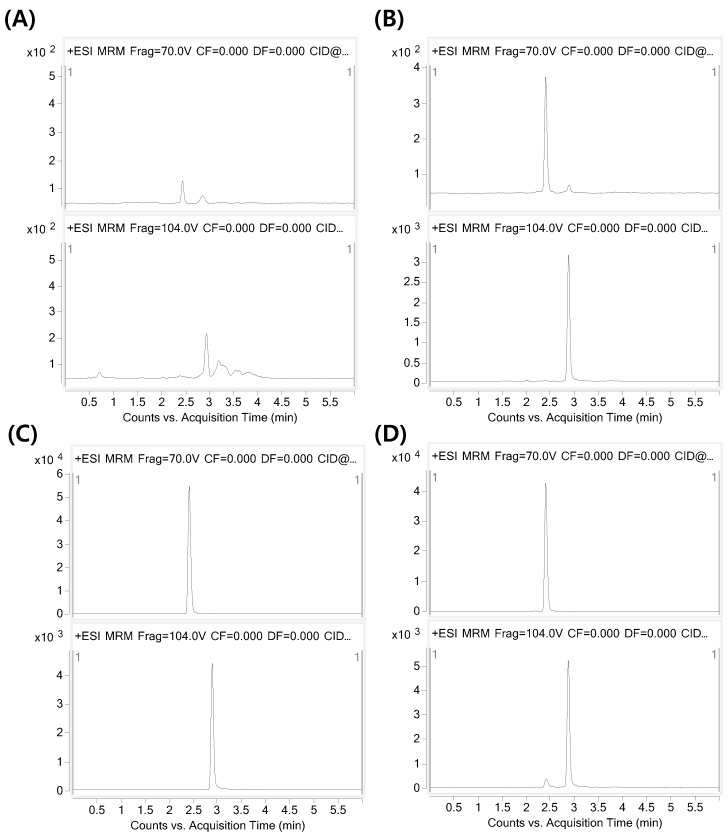
Typical MRM chromatograms of ATEC (top) and TBC (IS; bottom), (**A**) blank rat plasma, (**B**) blank rat plasma spiked with ATEC (10 ng/mL) and IS (100 ng/mL); rat plasma samples taken (**C**) 2 min after i.v. and (**D**) 15 min after p.o. administration of ATEC.

Representative multiple reaction monitoring chromatograms are shown in Figure 2A,B for blank plasma and plasma spiked with ATEC (10 ng/mL) and TBC (100 ng/mL). Chromatograms of the plasma samples obtained 1 h after i.v. and p.o. administration of ATEC to the rats are illustrated in Figure 2C,D. Sharp and fine peaks were obtained at retention times of 2.4 and 2.9 min for ATEC and TBC (IS), respectively. No interference was observed, indicating that the developed method was selective for the analytes in the plasma samples. 

#### 3.1.2. Linearity 

Seven calibration standards (i.e., 10, 50, 100, 200, 500, 1000, and 2000 ng/mL) of ATEC were used for construction of the plasma calibration curve. The calibration standard curves were linear and reliable over the standard concentrations across the calibration range. The regression coefficient (*r*^2^) was greater than 0.990. The precision and accuracy of the calibration standard curves were reliable (less than ± 15% RSD) for all the tested concentrations.

#### 3.1.3. Precision and Accuracy 

The QC samples were assayed for intra-day and inter-day precision and accuracy at concentrations of 10, 30, 300, and 1600 ng/mL (Table 1). The intra-day and inter-day accuracies were 89.1–102.9% and 94.0–99.8%, respectively. Intra-day and inter-day precisions were within 8.0% and 8.2%, respectively.

#### 3.1.4. Extraction recovery, matrix effect, and process efficiency

At concentrations of 50 and 1600 ng/mL, the mean extraction recoveries of ATEC from rat plasma were 103.9 ± 6.9% and 93.4 ± 6.5%, respectively, and the matrix effects were 67 ± 4.0% and 62.9 ± 3.2%, respectively. The mean process efficiencies were 69.6 ± 4.3% and 58.9 ± 7.0% at concentrations of 50 and 1600 ng/mL, respectively. The data indicated that ATEC was easily recovered from rat plasma after protein precipitation, and that the matrix effect was negligibly low.

#### 3.1.5. Stability

The stability of ATEC was investigated in a standard stock solution and plasma samples at QC levels. The resulting data are summarized in Table 2. ATEC was stable in rat plasma samples stored at −20 °C for 14 days (>90.8%, *n* = 3), at room temperature for 8 h (>96.3%, *n* = 3), and during three cycles of freeze and thaw at −20 °C (>105.8%, *n* = 3). The postpreparative QC samples were stable in an auto-sampler at 4°C for at least 12 h (*n* = 3, >91.0%). These results indicated that ATEC was stable during all the preparation and analytical procedures.

### 3.2. Pharmacokinetic Study

**Figure 3:**
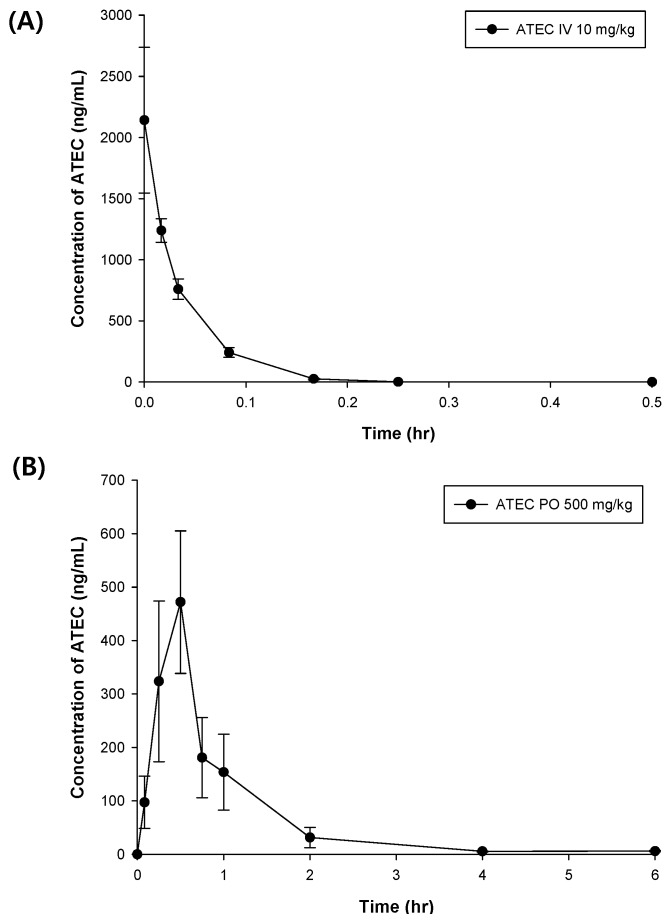
Mean plasma concentration–time profile of ATEC (A) after intravenous (i.v.) administration of 10 mg/kg of ATEC to rats (*n* = 6) and (B) after oral (p.o.) administration of 500 mg/kg (*n* = 6). Data are expressed as mean ± SD. The initial concentrations in the i.v. administration were estimated and indicated.

The developed method was successfully applied in a pharmacokinetic study of ATEC. We set the i.v. dose (10 mg/kg) according to the previous toxicology studies [6,7], but the oral pharmacokinetics was investigated at a higher dose (500 mg/kg), considering the detectability of ATEC. Figure 3 shows the mean plasma concentration–time profiles of ATEC following i.v. and oral administration. The plasma concentrations of ATEC rapidly decreased and were not detected after 0.5 h and 6 h following the administration of the i.v. dose of 10 mg/kg and the oral dose of 500 mg/kg of ATEC to the rats. The basic pharmacokinetic parameters of ATEC in the rats were calculated on the basis of the plasma concentration data represented in Table 3. The volume of distribution (Vz) was 4.8 L/kg, which was higher than the volume of body water, indicating the possibility of distribution into the extra-vascular space. The absolute bioavailability (F) of ATEC was 14.8% (as calculated by the dose-corrected AUC p.o. divided by the AUC i.v.). This relatively low F value pointed to poor absorption of ATEC from the gastrointestinal tract, degradation of ATEC in the gut, and extensive first-pass effects. After i.v. administration, ATEC was rapidly eliminated with a terminal half-life of 0.03 and mean residence time of 0.03 h, whereas oral administration of ATEC resulted in 1.03 h of terminal half-life and a mean residence time of 6.56 h. Longer terminal half-life and residence time were observed after oral administration than intravenous injection. It has been reported that this phenomenon is observed for certain drugs [21]. There could be several reasons, but one possible cause is flip-flop [21]. In this case, the absorption rate is much slower than the elimination rate, and the terminal slope is not determined by clearance and volume of distribution. Rather, bioavailability factors such as the rate and extent of absorption may control the terminal slope of the oral dose. This factor might affect the higher Vz/F value of the oral dose [22].

### 3.3. Metabolism Study

**Figure 4:**
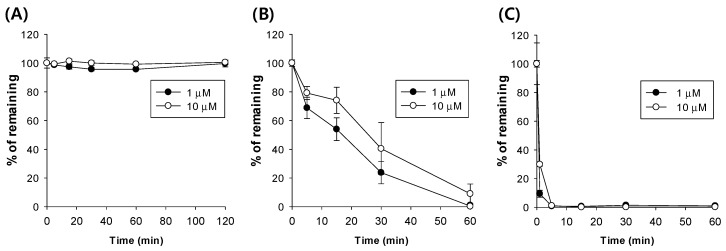
Metabolic stability of ATEC in (**A**) phosphate-buffered saline (pH 7.4), (**B**) rat plasma, and (**C**) rat liver microsomes.

To characterize the metabolism of ATEC, we tested the metabolic stability of ATEC in rat plasma and liver microsomes (Figure 4). ATEC was very stable in pH 7.4 phosphate buffered saline. In rat plasma, ATEC was gradually metabolized, and less than 20% of the initial amount remained 60 min after incubation. However, ATEC was rapidly metabolized in liver microsomes and readily disappeared in 5 min after incubation. These data suggested that ATEC may be eliminated mainly via metabolic clearance. 

**Figure 5:**
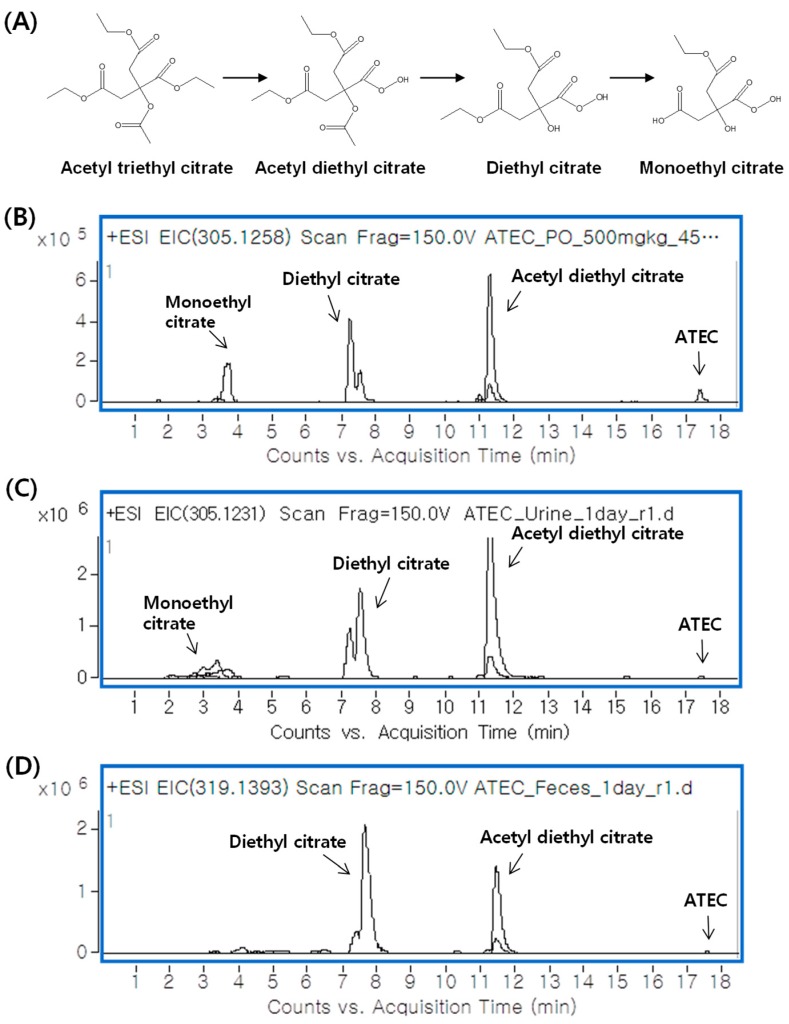
(**A**) Proposed metabolic pathway of ATEC and representative LC–MS chromatograms of (**B**) plasma, (**C**) urine, and (**D**) feces from rats that were orally administered ATEC.

Figure 5A depicts the postulated metabolic pathways of ATEC. The LC–QTOF MS analysis of plasma, urine, and feces samples showed that ATEC was extensively metabolized and excreted mainly as metabolites rather than as the parent form (Figure 5B–D). This finding was consistent with the in vitro metabolic stability data. The peaks of metabolites were postulated to correspond to acetyl diethyl citrate, diethyl citrate, and monoethyl citrate. The accurate mass data for the postulated metabolites are presented in Table 4. These data indicated that the major metabolic pathway of ATEC is via hydrolysis, mediated by esterase.

**Figure 6:**
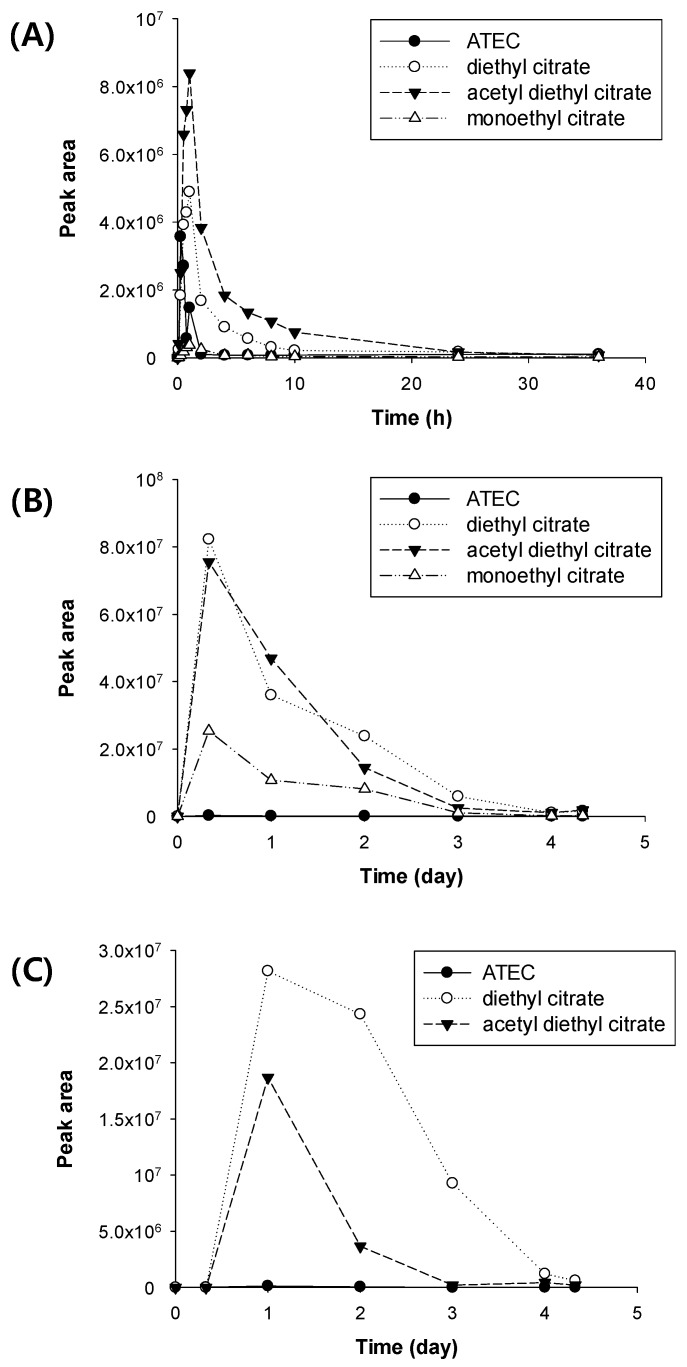
Plot of time–peak area for ATEC and its metabolites in (**A**) plasma, (**B**) urine, and (**C**) feces from the rats orally administered ATEC.

The metabolite profile according to time was plotted according to the peak area (Figure 6). Acetyl diethyl citrate and diethyl citrate were the major metabolites of ATEC. Unchanged ATEC was mostly excreted within 24 h after the administration of the dose, but acetyl diethyl citrate and diethyl citrate were excreted up to 4 days after the dose. 

Previously, we reported the pharmacokinetic properties of ATBC that is also a citrate plasticizer and has a similar structure to that of ATEC [23]. In terms of metabolic stability, these two citrate plasticizers showed weak stability for metabolism in plasma and liver microsomes. However, ATEC was more unstable in liver microsomes than ATBC, which may result in the lower oral bioavailability of ATEC (15.9%) compared with that of ATBC (27.4%) [23].

## 4. Conclusions

ATEC is used as a plasticizer in various pharmaceutical polymers. Although there have been some studies of the toxicity of ATEC, its biological properties in terms of absorption, metabolism, and excretion have not been reported. Furthermore, there have been no studies of analytical methods to determine ATEC in biological samples. In this study, we developed an accurate, sensitive, and reliable bioanalytical method for the determination of ATEC in rat plasma based on LC–MS/MS. The selectivity, linearity, precision, accuracy, and stability of the developed method were successfully validated and met established criteria. The developed method was subsequently applied to a pharmacokinetic study in rats. The pharmacokinetic study showed that ATEC rapidly disappeared from plasma. The metabolite profiling data indicated that ATEC was extensively metabolized and excreted mainly as metabolites rather than as the parent form. To our knowledge, this is the first report demonstrating a bioanalytical method for the determination of ATEC in biological samples and its pharmacokinetic properties. The resulting pharmacokinetic data can aid the understanding of the safety and toxicity of ATEC.

## Figures and Tables

**Table 1:** Intra- and inter-day accuracy and precision for the determination of ATEC in rat plasma. QC, Quality Control, CV, LLOQ, lower limit of quantification.

QC level	Nominal (ng/mL)	Intra-day (*n* = 5)		Inter-run (*n* = 3)	
		Accuracy (%)	CV (%)	Accuracy (%)	CV (%)
LLOQ	10	101.5	8.0	98.2	6.9
Low	30	102.9	6.8	98.2	3.3
Mid	300	100.7	4.5	99.8	2.6
High	1600	89.1	3.0	94.0	7.4

**Table 2:** Stability of ATEC in rat plasma.

Stability Test		% Recovery	
		50 ng/mL	1600 ng/mL
Short-term	(RT ^a^ for 8 h)	96.3 ± 3.6	97.9 ± 7.0
Long-term	(−20 °C for 21 day)	90.8 ± 6.1	97.3 ± 7.5
Freeze–thaw	(−20 °C, 3 cycles)	105.8 ± 8.7	111.6 ± 4.9
Post-preparative	(4 °C for 12 h)	95.1 ± 4.6	91.0 ± 4.3

^a^, room temperature.

**Table 3:** Pharmacokinetic parameters after i.v. and p.o. administration of ATBC to rats.

Parameter	IV (10 mg/kg, *n* = 6)		PO (500 mg/kg, *n* = 6)	
	GM	95% CI	GM	95% CI
AUC (ng·h/mL)	78.1	64.5–95.7	529.6	379.4–893.9
*T*_max_ (h) ^a^	-	-	0.5	0.25–0.5
*C*_max_ (ng/mL)	1219.6	1049.6–1427.6	444.5	271.8–834.3
*T*_1/2_ (h)	0.03	0.02–0.03	1.03	0.75–1.46
Vz (L/kg)	4.6	3.7–5.8	215.6 ^b^	8.1–733.2
Cl (L/h/kg)	125.1	105.0–150.6	145.5 ^c^	47.6–333.1
MRT (h)	0.03	0.03–0.04	6.56	3.58–12.63

GM: geometric mean; CI: confidence interval; AUC: area under the curve for concentrations; *T*_max_: time for peak concentration; Cmax: peak concentration; T_1/2_: elimination half-life; Cl: clearance; Vz: volume of distribution; MRT: mean residence time; F: bioavailability. ^a^ represented as a median value with range. ^b^ Vz/F; ^c^ Cl/F

**Table 4:** Accurate mass data for ATEC and its metabolites.

Name	RT	Proposed Elemental Composition [M+H]+	Exact Mass	Measured Mass	Error (ppm)
ATEC	17.4	C14H22O8	319.1387	319.1388	−0.3
Acetyl diethyl citrate	11.5	C12H18O8	291.1074	291.1075	−0.3
Diethyl citrate	7.7	C10H17O7	250.1047	250.1057	−4.0
Monoethyl citrate	3.4	C8H12O7	221.0656	221.0655	0.5
